# Acquired hemophagocytic syndrome in a patient with synovial sarcoma: a case report

**DOI:** 10.4155/fso.15.27

**Published:** 2015-11-01

**Authors:** Chiara Ciccarese, Roberto Ferrara, Emanuela Fantinel, Camilla Zecchetto, Francesca Simionato, Elisabetta Grego, Silvia Ortolani, Mario Caccese, Davide Bimbatti, Sara Cingarlini, Matteo Brunelli, Angelo Andreini, Giampaolo Tortora, Francesco Massari

**Affiliations:** 1 Medical Oncology, Azienda Ospedaliera Universitaria Integrata (A.O.U.I.), University of Verona, P.le L.A Scuro 10, 37134 Verona, Italy; 2 Department of Pathology & Diagnostic, Azienda Ospedaliera Universitaria Integrata (A.O.U.I.), University of Verona, Verona, Italy; 3 Department of Medicine, Section of Hematology & Bone Marrow Transplant Unit, Azienda Ospedaliera Universitaria Integrata (A.O.U.I.), University of Verona, Verona, Italy

**Keywords:** chemotherapy, hemophagocytic lymphohistiocytosis syndrome, synovial sarcoma

## Abstract

Hemophagocytic lymphohistiocytosis (HLH) is a syndrome characterized by severe hyperinflammation due to an overwhelming ineffective immune response to different triggers. Most important symptoms are fever, hepatosplenomegaly and cytopenias. Biochemical signs include elevated ferritin, hypertriglyceridemia and low fibrinogen. Hemophagocytosis in the bone marrow is a hallmark of this syndrome. Based on the pathogenetic mechanism, it can be classified into primary (inherited) or secondary (acquired) HLH. We report, to our knowledge, the first case of acquired hemophagocytic syndrome that arose in a 20-year-old man affected by synovial sarcoma as a complication during chemotherapy.

## Abstract (lay abstract)

The importance of this case report resides mainly in the difficulty of identifying a rare syndrome, as the hemophagocytic lymphohistiocytosis is, which can gather and justify several unspecific clinical and biochemical signs and symptoms. Hardly, prolonged fever, hepatosplenomegaly and a lasted cytopenia arising in a patients with solid tumor (a synovial sarcoma in our case) during chemotherapy, not directly attributable and not fully justifiable by infections, cytotoxic treatment or by the cancer itself, lead clinicians assuming the hemophagocytosis as a possible etiology. Therefore, describing a peculiar case, which promptly responded to corticosteroid therapy, can help to bring about an early diagnosis and set quickly an adequate treatment so as to change the patient's prognosis.

**Figure 1.**
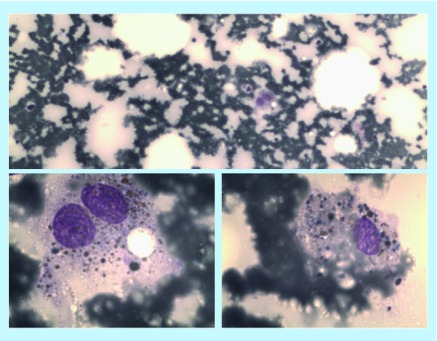
**Cytological evidence of hemophagocytosis.** Hemophagocytosis is clearly a ‘dynamic’ process of disruption on red blood cell by histiocytes. Hysto-cytological examination of the bone marrow is just a ‘static’ picture of a single phase of the hemophagocytic process. In this figure, the hemosiderin inside the macrophages represents the end stage of hemophagocytosis, when the external membrane of red blood cell has already been disrupted.

Hemophagocytic lymphohistiocytosis (HLH) is a life-threatening syndrome characterized by severe hyperinflammation due to an overwhelming ineffective immune response to different triggers [1]. Cardinal symptoms are fever, hepatosplenomegaly and cytopenias. Biochemical signs include elevated ferritin, hypertriglyceridemia and low fibrinogen. Hemophagocytosis in the bone marrow is a hallmark of this syndrome (Box 1). Based on the pathogenetic mechanism, it can be classified into primary (inherited) or secondary (acquired) HLH [2]. Impaired function of NK cells and macrophages, as well as cytotoxic T lymphocytes deficiency, characterizes all forms of HLH. Genetic mutations leading to primary HLH impair the granule-dependent cytotoxic activity of lymphocytes [3]. Secondary forms are described in association with infections, autoimmune disorders, acquired immunodeficiency and malignancies, mainly lymphoproliferative diseases [4,5]. Solid tumors are seldom related to HLH [6–9]. Treatment should be started promptly in order to suppress the hyperinflammatory status. Steroids, immune-suppressive agents, cytotoxic drugs, biological response modifiers and stem-cell transplantation are available strategies, however, far from being curative in most cases [10].

To our knowledge, we report the first case of acquired hemophagocytic syndrome arose in a 20-year-old man affected by synovial sarcoma as a complication during chemotherapy. We hypothesize that an abnormal neoplastic production of cytokines, undetected infection, the iatrogenic immunosuppression status or the direct cytolytic effect of chemotherapy may have been the precipitating cofactor.

## Case presentation

A 20-year-old man, affected by synovial sarcoma of the left buttock with pulmonary metastases since October 2013, was treated with doxorubicin and ifosfamide as first-line chemotherapy. After two courses of doxorubicin and ifosfamide, complicated by febrile neutropenia despite primary prophylactic G-CSF support but with a partial response of the disease on CT scan revaluation, the patient received single-agent chemotherapy with high-dose ifosfamide (14 g/m^2^ continuous infusion for 14 days). The 13th day of ifosfamide infusion the patient was hospitalized for fever and severe mucositis and diarrhea (grade 4 according to CTCAE v4.03).

Laboratory tests revealed low counts in all blood cell types; the hemoglobin nadir was 6.6 g/dl, the white blood cell count was 180/mm^c^, with severe neutropenia (absolute neutrophil count <10), and the platelet nadir was 5000/mm^c^. Furthermore, the C-reactive protein (PCR) was significantly augmented, without a concomitant rise of procalcitonin. The serum ferritin level was increased to 19,500 ng/ml. The clinical examination showed the presence of hepatomegaly, with no laboratory evidence of hepatic dysfunction, a maculopapular erythematous rash spread from the trunk to the extremities, and a severe oral mucositis. Lymphadenopathies were not detectable.

At the beginning, the pancytopenia was ascribed to chemotherapy myelotoxicity. The patient was therefore supported with periodic packed red blood cells and platelets transfusions and with G-CSF. Considering the presence of febrile neutropenia, possible sources of infections were excluded through a chest X-ray, urine cultures, blood cultures from central venous catheter and peripheral vein. In any case, a broad-spectrum antibiotic therapy was set up, initially with piperacillin/tazobactam, daptomycin and, given the diarrhea, metronidazole. The persistence of fever and the appearance of a parenchymal lung lesion at a monitoring chest x-ray moved us to replace antibiotic therapy with meropenem, vancomycin and levofloxacin, with no clinical improvement nor defervescence.

Fifteen days after admission, fever and pancytopenia still persisted. The sole chemotherapy could not be the only cause. A bone marrow aspiration and biopsy were performed, with cytological evidence of hemophagocytosis (Figure 1). The histologic pattern confirmed the presence of bone marrow aplasia, with rare macrophages containing hemosiderin deposits. Neoplastic bone marrow infiltration was excluded.

The patient received intravenous gammaglobulin infusions for immunomodulatory purposes, with insignificant effects on hematopoiesis.

Collaterally, due to the prolonged neutropenia, antiviral (acyclovir) and antifungal (posaconazole) therapies were introduced as prophylaxis. There were no findings of active viral infection (i.e., Cytomegalovirus – IgM and IgG antibodies and PRC, Epstein-Barr virus - viral capsid antigens (VCA) IgG, VCA IgM, and EBV nuclear antigen (EBNA)-1 IgG antibodies, Parvovirus B19 - anti-B19 IgM and IgG antibodies with western blot and B19 DNA with PCR, Herpesvirus-6 and 7- with PCR) that could justify the bone marrow aplasia. The patient's clinical condition worsened. Although the state of consciousness of the patient was never altered, even during peaks of fever, appeared persistent cough and dyspnea for modest efforts. Moreover, 18 days after admission, the serum positivization assay of beta-D-glucan and Aspergillus antigen suggested the onset of a fungal infection. Therefore, the pulmonary status was investigated through a high-resolution CT scan, with evidence of a large lung cavity resembling the typical ‘fungus ball’. Thus, the patient started antifungal therapy with amphotericin B.

At this point, the main problem was the assessment of the fine balance between the potential benefit of steroid therapy on hematopoiesis and its detrimental effect on fungal infection. Considering the resumption of the bone marrow activity as a priority, for both the magnitude of blood transfusion needs and its crucial role in facilitating the lung infection resolution, we started corticosteroid therapy (dexamethasone 8 mg intravenously). Surprisingly, 2 days after corticosteroid infusions white blood cells began to rise, reaching normal values after 6 days of steroid therapy. The platelet count showed a trend of slower growth.

The antifungal therapy in combination with the resumption of the immune system efficacy resulted in a progressive clinical, laboratoristic and radiological resolution of the infection. After 2 months, the patient was discharged.

To our knowledge this is the first case of acquired HLH in a patient with synovial sarcoma during chemotherapy treatment.

Which was the precipitating factor in the case reported is difficult to define. Probably, the coexistence of an overwhelming neoplastic production of cytokines, an unrecognized fungal infection, the iatrogenic immune suppression due to chemotherapy or a direct cytolytic effect of chemotherapy could have contributed.

Ifosfamide is a structural synthetic analog of cyclophosphamide, and thus a nitrogen mustard-like alkylating agent. After hepatic microsomal activation, biologically active alkylated metabolites interact with DNA to form covalent bonds. Myelosuppression is dose-related and dose-limiting. It consists primarily in leukopenia and, to a lesser extent, piastrinopenia. At ifosfamide dosage of 10–12 g/m^2^/cycle, leukopenia is almost the rule. Myelosuppression is usually reversible, the nadir is reached after 8–14 days, with recovery in 18–20 days. In the present case, the persistent severe pancytopenia with no evidence of bone marrow recovery could not be ascribed exclusively to the myelosuppressive effect of ifosfamide. Alkylating agent-induced tumor cell lysis could instead have stimulated an immune response, resulting in HLH.

In our case, the clinical suspicion of an incoming complication that would justify the prolonged pancytopenia led to the diagnosis. Cytopenia, persistent fever, hepatomegaly, high levels of ferritin and the histological finding of hemophagocytosis were therefore ascribed to peculiar manifestations of acquired HLH.

As concerns the treatment, the peculiarity of our case also depends on the rapid onset of response to monotherapy with corticosteroids. Luckily, the use of immunosuppressive drugs was not necessary, avoiding therefore to aggravate active fungal pulmonary infection.

## Conclusion

In conclusion, we report a noteworthy case of HLH secondary to chemotherapy for synovial sarcoma that achieved prompt remission with steroids, highlighting the importance to recognize a, albeit rare, correlation between HLH and solid tumors.

Box 1. **Diagnostic criteria for hemophagocytic lymphohistiocytosis used in the HLH-2004 trial^†^.**

A. Molecular diagnosis consistent with HLH: pathologic mutations of PRF1, UNC13D, Munc188–2, Rab27a, STX11, SH2D1A or BIRC4 or
B. Five of the eight criteria listed below are fulfilled:
  Fever ≥38.5°C
  Hepatosplenomegaly
  Cytopenias (affecting at least two of three lineages in the peripheral blood)
    Hemoglobin <9 g/dl (in infants <4 weeks: hemoglobin <10 g/dl)
    Platelets <100 × 10^3^/ml
    Neutrophils <1 × 10^3^/ml
  Hypertriglyceridemia (fasting, >265 mg/dl) and/or hypofibrinogenemia (<150 mg/dl)
  Hemophagocytosis in bone marrow, spleen, lymph nodes or liver
  Low or absent NK-cell activity
  Ferritin >500 ng/ml^‡^
  Elevated s CD25 (soluble IL-2 receptor α-chain)

^†^Adapted from [11].
^‡^Although the HLH-2004 protocol uses ferritin >500 ng/ml, ferritin >3000 ng/ml is suspect for HLH, and ferritin >10,000 ng/ml highly related to HLH [12].HLH: Hemophagocytic lymphohistiocytosis.

**Executive summary**

Hemophagocytic lymphohistiocytosis (HLH) is a rare, life-threatening, hyperinflammatory syndrome, peculiarly characterized by hemophagocytosis in the bone marrow, and clinically marked by fever, hepatosplenomegaly and pancytopenia.
Inhered and acquired forms can be distinguished. Different triggers have been identified as possible etiologic agents, seldom including solid tumors.
We reported the first case of acquired HLH in a patient with synovial sarcoma during chemotherapy treatment.
The neoplastic production of cytokines, an unrecognized fungal infection, the iatrogenic immune suppression due to chemotherapy, or a direct cytolytic effect of chemotherapy can be listed as contributing etiological factors.
The peculiarity of our case also depends on the rapid onset of response to monotherapy with corticosteroids.
